# Complete Genome Sequence of Foot-and-Mouth Disease Virus Type A Circulating in Bangladesh

**DOI:** 10.1128/genomeA.00506-14

**Published:** 2014-06-12

**Authors:** Huzzat Ullah, Mohammad Anwar Siddique, Munawar Sultana, M. Anwar Hossain

**Affiliations:** Department of Microbiology, University of Dhaka, Dhaka, Bangladesh

## Abstract

The complete genome sequence of a foot-and-and mouth disease virus (FMDV) type A strain (BAN/GA/Sa-197/2013), isolated from Gazipur in Bangladesh, revealed an 84-nucleotide insertion within the 5′-untranslated region (UTR), a lengthened poly(C) tract, and amino acid substitutions at the VP1 region compared to the available genome sequence of the vaccine strain (GenBank accession no. HM854025).

## GENOME ANNOUNCEMENT

Foot-and-mouth disease virus (FMDV), a single-stranded positive-sense RNA virus belonging to the *Picornaviridae* family and genus *Aphthovirus*, is highly infectious and causes disease in domestic and wild cloven-hoofed animals. The characteristic feature of FMDV, like other RNA viruses, is that it is genetically diverse and includes seven distinct serotypes (O, A, C, Asia-1, and SAT 1 to 3) and multiple subtypes worldwide (1). FMDV serotype A is grouped into the topotypes Asia, Europe-South America, and Africa based on genotyping, and it seems to be the most genetically and antigenically diverse among all FMDV serotypes (2, 3). In Bangladesh, serotype A, belonging to genotype VII of the Asia topotype viruses, was detected to be endemic across the country (4).

Here, we report the complete nucleotide sequence of an FMDV type A strain (BAN/GA/Sa-197/2013) isolated from the tongue epithelium of ruptured vesicles of an infected cow and collected on 25 December 2013 in Gazipur, Bangladesh. The viral RNA, extracted directly from the infected cell culture supernatant at passage 3 in a BHK-21 cell line, was reverse transcribed using random and oligo(dT) primers for cDNA synthesis. Using internal primer pairs, 22 overlapping amplicons spanning the entire viral genome were generated, sequenced (with an ABI genetic analyzer), and assembled into a whole genome using SeqMan version 7.0 (DNAStar Lasergene, USA). Phylogenetic analysis was carried out using the MEGA 5.2 software.

The genome of strain BAN/GA/Sa-197/2013 is 8,220 nucleotides (nt) in length and contains a 1,100-nt 5′-untranslated region (UTR) with a 17-residue poly(C) tract, a 6,999-nt single open reading frame coding for a polyprotein containing four structural proteins (VP4, VP2, VP3, and VP1) and eight nonstructural proteins (L, 2A, 2B, 2C, 3A, 3B, 3C, and 3D), and a 121-nt 3′-UTR with a 28-nt poly(A) tail. Phylogenetically, the isolated FMDV type A strain (BAN/GA/Sa-197/2013) clustered within genotype VII of the Asia topotype. BAN/GA/Sa-197/2013 shares 94% nucleotide homology (in relation to the complete genome) with IND 245/2007 (GenBank accession no. HQ832590).

A sequence comparison between BAN/GA/Sa-197/2013 and a currently available vaccine strain (accession no. HM854025) in Bangladesh and neighboring countries showed 10% variation at the nucleotide sequence level and 5% variation at the deduced amino acids of the VP region. Specifically, there are 15 substitutions in the VP1 region, including 4 amino acids (T44N, T45A, N46S, and T48I) in the B-C loop (residues 40 to 60) and 2 amino acids (T143V and I154V) in the G-H loop (residues 138 to 154), which indicates antigenic heterogeneity. Furthermore, there is an 84-nt insertion at positions 394 to 477 within a region of the 5′-UTR and a 2-nt insertion at the 3′-UTR in comparison to the vaccine strain.

We report here the first complete genome sequence data for a local strain of FMDV type A isolated from cattle in Bangladesh. The complete genetic information may be helpful to select the suitable vaccine candidate against the FMD prevailing in Bangladesh.

### Nucleotide sequence accession number. 

The complete genome sequence of FMDV isolate BAN/GA/Sa-197/2013 has been deposited in GenBank under the accession no. KJ754939.
